# Landscape analysis of pharmacovigilance and related practices among 34 vaccine manufacturers’ from emerging countries

**DOI:** 10.1016/j.vaccine.2020.06.016

**Published:** 2020-07-22

**Authors:** Katharina Hartmann, Sonia Pagliusi, Alexander Precioso

**Affiliations:** a Independent Consultant, Kusnacht, Switzerland; b DCVMN International, Route de Crassier 7, 1262 Nyon, Switzerland; c Butantan Institute, Av. Vital Brazil, 1500, 05503-900 Sao Paulo, Brazil

**Keywords:** Safety, Pharmacovigilance, Post-marketing surveillance, Vaccination, Infectious diseases

## Abstract

Developing Countries’ Vaccine Manufacturers Network was tasked with the strategic goal of seeking solutions, jointly with manufacturers, for enabling the stable, sustainable supply of quality vaccines to developing countries to increase global immunization. As vaccines are given to millions of healthy people, including children, to prevent life-threatening diseases, vaccines must meet high safety standards. Vaccine safety monitoring is of paramount importance to maintain trust in vaccination programs globally. Once a vaccine is licensed and recommended for use, its safety and effectiveness must be monitored during its whole lifecycle, as the safety profile and protective effectiveness may change over time. A well-established safety governance model across the organization with underlying processes for data collection, signal and risk management and communication is essential. A “fit for purpose” pharmacovigilance system may vary as it depends on several factors. However, all vaccine manufacturers strive to achieve a pharmacovigilance system satisfying Good Pharmacovigilance Practices, in compliance with national, international and supranational requirements, as applicable.

A landscape analysis, using a questionnaire covering nine pharmacovigilance key areas related to an effective system, was conducted to understand the existing pharmacovigilance structures, practices and expertise of vaccine manufacturers from emerging countries, on an institutional level. 34 of the 43 contacted manufacturers participated voluntarily. The survey results show that all respondents have established vaccine safety capacity, mainly in collecting and handling adverse events following immunization and implementing standardized processes; the survey also shows differences in the maturity of the manufacturers’ pharmacovigilance system, Quality Management System, signal and risk management, and safety governance. The analysis provides a tool for manufacturers to gain a “bird's-eye” view of the structure of pharmacovigilance key areas and the operational dimensions covered by each area, to benchmarking against international expectations, serving as a basis to further strengthen pharmacovigilance systems, to support accelerated global vaccine supply.

## 1 Introduction

Recent years revealed the spread of emerging and re-emerging outbreaks such as Ebola, Middle East Respiratory Syndrome (MERS), yellow fever, polio, influenza, measles, dengue and Zika, as well as shortages in vaccines such as Bacillus Calmette-Guérin (BCG), Tetanus-Diphtheria-Pertussis (DTP), human papillomavirus. Vaccines against such infections, if available, can be given to millions of healthy people, including children, to prevent life-threatening diseases, and they are assessed against very high safety standards. Thus, vaccine safety monitoring is of utmost importance to maintain a high level of safety, quality, and coverage and trust in vaccination programs [1]. Following clinical studies to demonstrate safety, tolerability, immunogenicity and efficacy, once a vaccine is licensed and recommended for use, its safety must be monitored during its whole product lifecycle, as the safety profile as well as protective effectiveness may evolve over time [2], [3].

Pharmacovigilance (PV) is a crucial component in vaccine safety monitoring and for all stakeholders involved in developing or providing vaccines to the public, vaccine safety is a key responsibility [4], [5]. Safety monitoring by implementing a high-quality PV System is also a priority for vaccine manufacturers. Thorough understanding of vaccine safety key performance indicators and accurate monitoring tools enable manufacturers to accelerate registrations and establish dynamic processes to analyze an evolving benefit/risk profile for product development.

A well-established safety governance model across the organization, with solid underlying processes for signal and risk management as well as risk communication, may indicate the maturity of the manufacturer’s PV system. A PV System needs to be “fit for purpose”, depending on a number of factors, e.g. number of vaccines in the portfolio, number of market authorizations, World Health Organization (WHO) pre-qualification status, adoption in public immunization programs, number of doses distributed, nature of licensor and/or licensee of vaccines, number of vaccines in development and respective development stage, size of the organization, country regulations, to name a few. Thus, also in developing countries, all manufacturers and market authorization holders strive for Good Pharmacovigilance Practice (GPvP) [6], [7] to achieve a system that satisfies compliance with national as well as with international (e.g., International Council for Harmonisation, ICH; Food and Drug Administration, FDA; European Medicines Agency, EMA) or supranational (e.g., WHO, Pan Americn Health Organization, PAHO) requirements. Mature PV systems should be able to reach accurate assessments despite diverging regulatory requirements and vaccine manufacturers in countries where ICH standards are not (yet) adopted and implemented in the legislation could consider establishing a PV system aligned with international standards.

Adverse events following immunization (AEFIs) or a potential safety concern, which occurs in the context of a product quality problem, needs to be analyzed by PV in collaboration with manufacturer, supplier or distributor, as appropriate, to assess the safety risk and the risk of harm to vaccinees exposed to the detected product quality problem; such analyses may result in a Health Hazard Evaluation (HHE).

In seeking solutions to scale up global immunization, improving access to vaccines, and enhancing scientific knowledge and operational efficiency in PV, for the stable and sustainable supply of quality vaccines, the Developing Countries’ Vaccine Manufacturers Network (DCVMN)^1^ conducted a survey among its members that was presented and discussed in a meeting. The level of PV practices among vaccine manufacturers in emerging countries was assessed through a survey, in a certain point in time, and the results shed light on specific areas for strengthening manufacturers’ PV systems and are reported here.

## 2 Working methodology and process

To understand existing PV structures, practices and expertise among developing countries’ vaccine manufacturers, a cross-sectional survey was set up using a questionnaire covering nine areas and important aspects of PV as per ICH Guidelines and European Medicines Agency (EMA) “Good Vigilance Practice”: pharmacovigilance structure, collection and handling of AEFIs from spontaneous/passive reporting as well as from active collection (e.g., clinical trials and other sources), aggregate reports, safety database systems, pharmacovigilance quality management systems, safety signal and risk management including communication and business agreements concerning the exchange of safety data. The questionnaire was designed by a PV expert in consultation with the DCVMN International Secretariat, to ensure assessed topics were relevant for vaccine manufacturers in emerging countries. The survey covering nine PV key functional areas was composed of 67 questions; responses to questions could only be YES or NO. The survey was created on 14 March 2019 and verified for functionality by DCVMN secretariat. All 43 vaccine manufacturers’ members of the Network, at that time, were invited, by email on 25 March 2019, to voluntarily reply to the questions, whereby responses were anonymous. Reminders were sent by email on 09, 19 and 26 April 2019. The last response was received on 23 May 2019. Results were collected by DCVMN Secretariat and shared with the expert consultant in an anonymous manner for independent analysis. Percentage of positive responses was the guiding parameter. The desk assessment of each area was conducted by the expert consultant and focused on qualitative and descriptive analysis; thus, it was not relevant to conduct any statistical analysis at this stage. The number and content of the documented processes (e.g., SOPs, policies, working instructions, guidelines) may vary depending on PV activities performed, and were not assessed in this analysis. It was assumed that in mature Pharmacovigilance structures it could be expected that the “YES” answer rate for all PV activities would be around 80%, and the survey was used to analyze needs; thus, a low proportion of YES responses indicates need for strengthening. Although, there are many national, cultural and regulatory differences among countries impacting PV implementation, these aspects were not assessed. Data on size of organization, business type (public/private), geographical location, and specific vaccines types produced were not asked to respect the confidentiality of the survey.

The results of this work were presented to a group of PV experts from DCVMN affiliated vaccine manufacturers from 9 companies from 7 countries (Argentina, Brazil, China, India, Indonesia, Thailand, Vietnam), in an informal workshop held in Sao Paulo, on 27th to 30th May 2019, where the participants reviewed the outcome of the comparative analysis for each of the PV areas and made corrections and adjustments, discussed the differences between practices worldwide and agreed on the training needs required in general. Group discussions served to validate the collected data, and the common needs across manufacturers identified through the survey.

## 3 Areas assessed by the survey

**Fig. 1:**
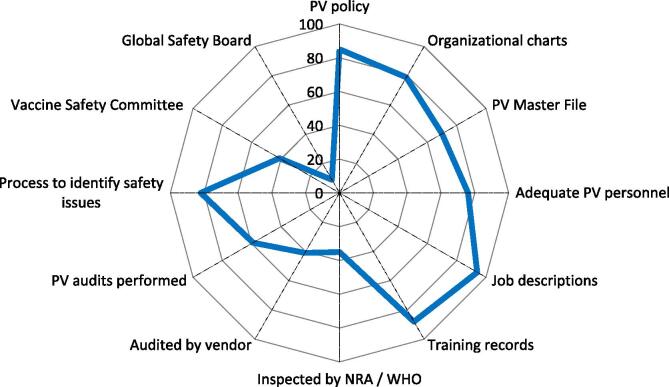
Corporate structure relating to PV. Spider diagram with 12 axis, each representing the percentage (from 0 to 100%) of YES answers (blue line) referring to «availability» of or «process/procedure in place», among the 34 corporate responders. Each axis reflects the availability or level of implementation of 12 specific activities relating to PV structures, in clockwise direction. NRAs = National Regulatory Authorities; WHO = World Health Organization. (For interpretation of the references to colour in this figure legend, the reader is referred to the web version of this article.)

**Fig. 2:**
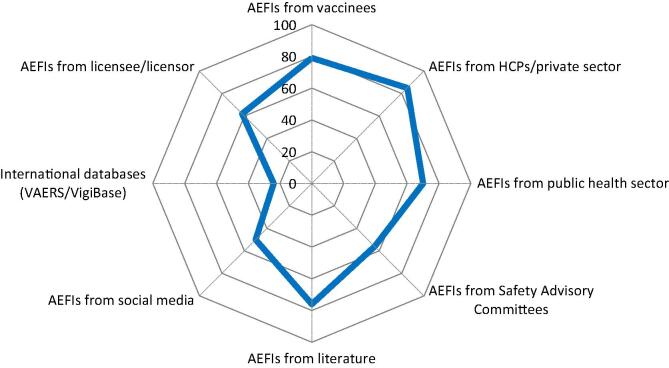
Collection of AEFIs from passive reporting. Spider diagram with 8 axis, each representing the percentage (from 0 to 100%) of YES answers (blue line) referring to the source of passively reported/collected AEFIs. AEFIs = Adverse Event Following Immunizations; VAERS = Vaccine Adverse Event Reporting System. (For interpretation of the references to colour in this figure legend, the reader is referred to the web version of this article.)

**Fig. 3:**
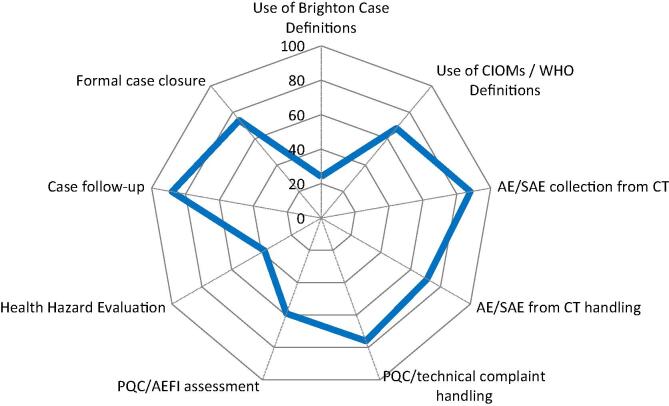
Collection and handling of AEFIs from active safety monitoring. Spider diagram with 9 axis, each representing the percentage (from 0 to 100%) of YES answers (blue line) referring to the 9 common practices related to active collection of AEFIs and their respective handling using documented procedures (SOPs). CIOMS = Council for International Organizations of Medical Sciences; AE/SAE = Adverse Event/Serious Adverse Event; PCQ = Product Quality Complaint. (For interpretation of the references to colour in this figure legend, the reader is referred to the web version of this article.)

**Fig. 4:**
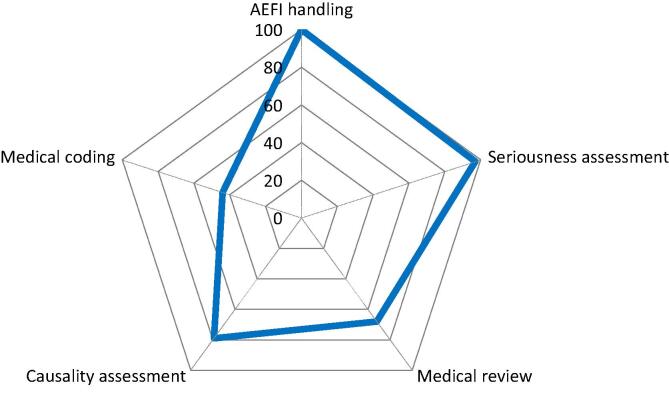
Management of AEFIs. Spider diagram with 5 axis, each representing the percentage (from 0 to 100%) of YES answers (blue line) to 5 questions referring to the availability of documented standard operating procedures (SOPs) for specific PV Adverse Events Following Immunization (AEFI) management. (For interpretation of the references to colour in this figure legend, the reader is referred to the web version of this article.)

**Fig. 5:**
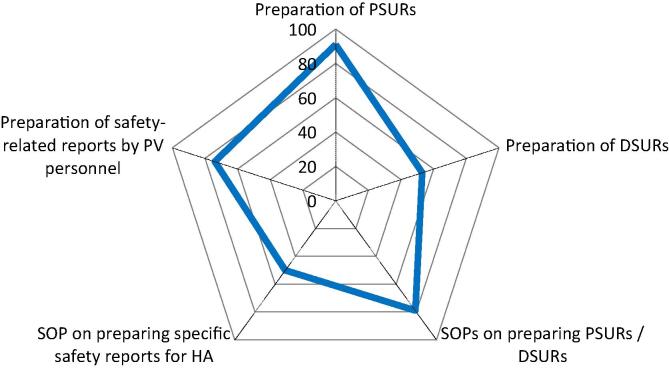
Management of aggregate periodic reporting (PSURs), annual reports, DSURs and Safety-related requests from Regulatory Bodies. Spider diagram with 5 axis, each representing the percentage (from 0 to 100%) of YES answers (blue line) to 5 questions referring to activities and the availability of respective documented standard operating procedures (SOPs) necessary to manage the preparation of aggregate periodic reports to regulatory bodies. DSURs = Development Safety Update Report; HA = Health Authority; PSUR = Periodic Safety Update Report. (For interpretation of the references to colour in this figure legend, the reader is referred to the web version of this article.)

**Fig. 6:**
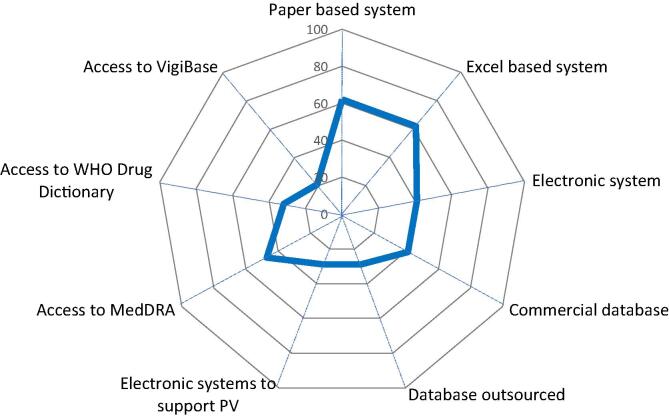
Safety Database Systems. Spider diagram with 9 axis, each representing the percentage (from 0 to 100%) of YES answers (blue line) to 9 questions referring to the availability of company’s safety databases and access to other PV supporting systems. (For interpretation of the references to colour in this figure legend, the reader is referred to the web version of this article.)

**Fig. 7:**
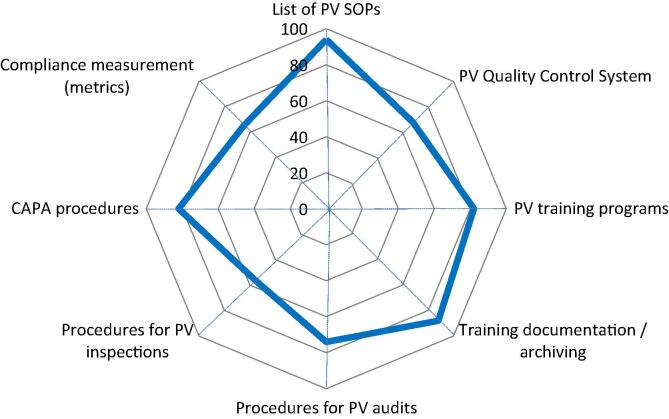
Pharmacovigilance Quality Management System. Spider diagram with 8 axis, each representing the percentage (from 0 to 100%) of YES answers (blue line) to 8 questions referring to the availability of respective documented processes/procedures for PV quality management. CAPA = Corrective And Preventive Actions. (For interpretation of the references to colour in this figure legend, the reader is referred to the web version of this article.)

**Fig. 8:**
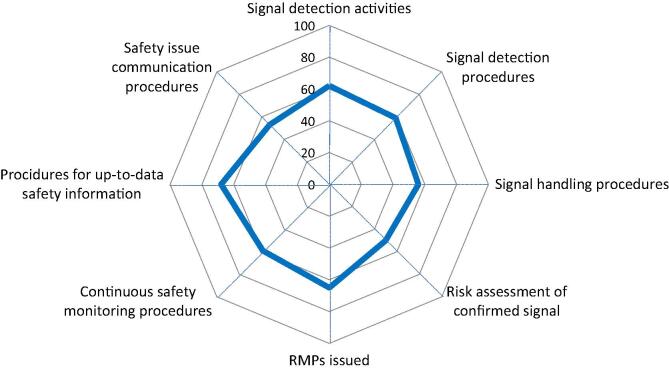
Safety Signal, Risk Management, and Safety Communication. Spider diagram with 8 axis, each representing the percentage (from 0 to 100%) of YES answers (blue line) to 8 questions referring to specific safety signal, risk management and safety communication activities and their respective documented standard operating processes/procedures. RMPs = Risk Management Plans. (For interpretation of the references to colour in this figure legend, the reader is referred to the web version of this article.)

**Fig. 9:**
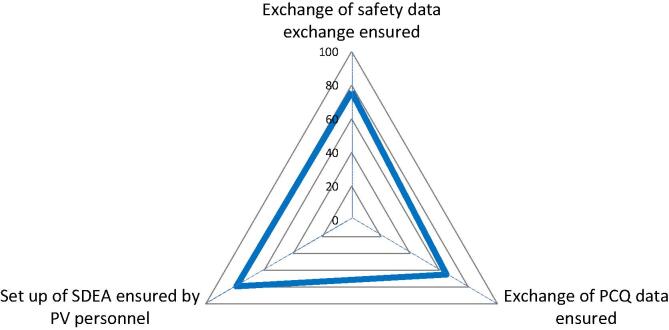
Pharmacovigilance Agreements (Safety Data Exchange Agreements, SDEA). Spider diagram with 3 axis, each representing the percentage (from 0 to 100%) of YES answers (blue line) to 3 questions referring to specific documented standard operating processes/procedures for safety data exchange in business agreements, between licensees/licensors. PCQ = Product Quality Complaint. (For interpretation of the references to colour in this figure legend, the reader is referred to the web version of this article.)

A questionnaire on nine key PV activities was circulated to the 43 vaccine manufacturing companies members of DCVMN, and 34 members (80% participation), based in 13 countries/territories,^2^ voluntarily responded to the survey in an anonymous manner. The overall results of the YES answers related to the nine key PV areas assessed by the different questions are shown in Table 1 and the results in percentages of the YES answers to each activity assessed per question are shown in Fig. 1, Fig. 2, Fig. 3, Fig. 4, Fig. 5, Fig. 6, Fig. 7, Fig. 8, Fig. 9.

**Table 1:** Overall pooled number of responses per category area.

Pharmacovigilance Activity per Category	Number of questions per category	Pooled number of YES answers per category	Pooled number of NO answers per category	Pooled number of answers YES + NO	Percentage of YES/total
Organizational Structure relating to PV	12	259	149	408	63%
AEFI collection from passive reporting	8	171	101	272	63%
AEFI collection from active reporting	9	198	108	306	65%
Management of AEFI	5	132	38	170	78%
Management of Aggregate Reporting	5	118	52	170	69%
Safety Database System	9	124	182	306	41%
PV Quality Management System	8	207	65	272	76%
Safety Signal and Risk Management, and Communication	8	160	112	272	59%
Safety Data Exchange Agreements	3	73	29	102	72%
Total	67	1442	836	2278	63%

This table displays the survey results of the nine PV key activities assessed: The first column indicates the nine different PV key categories; the second column shows the number of questions per category; the third column, the total number of YES responses reported per category, indicating that the respective activities and processes are performed (without any further specification on the level of implementation); the forth column, the total number of NO responses reported per category, indicating that the respective activities/processes are not performed; the fifth column shows the total number of all responses (YES and NO) per category; and the sixth column displays the percentage of YES responses per category.

### 3.1 Organizational structure relating to pharmacovigilance

The first area with the sub-questions, related to PV policy, PV System description, Organizational Charts, Resources, Training, Audits and Inspections, and Safety Governance, focused on assessing the structure of their PV System. A Pharmacovigilance Policy is not a mandatory regulatory document; still, 85% of respondents reported having a PV policy in place (Fig. 1). Examples of corporate PV policies are openly available [8], [9]. 79% of the respondents have organizational charts readily available. Such charts illustrate the relationships and the rank of PV function within the organization [10]. About 70% of the respondents indicated to have a Pharmacovigilance System Master File (PSMF) or a Detailed Description of the Pharmacovigilance System (DDPS) similar to the EU Pharmacovigilance System Master File (EMA GVP Module II) [11]. Assessment of personnel resources shows that over 75% of the respondents have at least three dedicated persons formally responsible for PV and 94% have up-to-date written job descriptions; 88% have up-to-date training records readily available. 35% of the manufacturers were inspected by a National Regulatory Authority (NRA) or by WHO and might show a higher level of implemented PV regulations and a better corporate understanding of the importance of a functioning PV System at Senior Management level. Indeed, 35% of the members’ manufacturers have WHO prequalified vaccines^3^, and NRA inspection of PV systems may be uncommon in emerging countries. External Audits, done for due diligence when entering into a business partnership or before outsourcing or during the outsourced business process, showed that 41% of the respondents indicated that they have been audited by an external vendor. Internal PV audits (“self-inspection”), to detect deviations or deficiencies of processes, and to check interactions between the different stakeholders within the organization with regard to product and vaccinee safety, showed that 59% of the respondents perform internal audits. Safety Governance refers generally to the escalation process of safety related issues within an organization and 82% of the respondents indicated that such safety governance processes are in place. An internal Vaccine Safety Management Committee (Safety Management Teams SMTs/Vaccine Safety Teams VSTs) is a cross-functional multidisciplinary team including e.g., PV, Regulatory Affairs, Manufacturing (CMC), Clinical Development, Quality Assurance, Epidemiology, Toxicology and other functions, and appears to be in place in 41% of the respondents. An established Global Safety Board reflects the manufacturers’ internal high-level commitment for safety governance and 9% of the respondents indicated have an operating Global Safety Board.

### 3.2 Collection of Adverse EventsFollowing immunizatios (AEFIs) from passive (spontaneous) reporting

79% of the respondents have established channels to receive spontaneously reported AEFIs directly from the public (vaccinees) (e.g., AEFI report forms available on the manufacturer’s website, other specific reporting lines for consumers/vaccinees) (Fig. 2). 85% of the respondents receive spontaneously reported AEFIs from health care providers (HCPs) from the private sector (e.g., from doctors, nurses, vaccine providers, hospitals, clinics etc.). 70% of the respondents receive spontaneously reported AEFIs from national vaccination campaigns or immunization programs and health facilities at national or regional level. Only 56% of the responders receive spontaneously reported AEFIs from national vaccine safety advisory committees, the national/supranational committees that monitor, investigate and respond to AEFIs. More than 75% of the responders perform medical literature screening for AEFIs. It remains to be established if they perform the screening regularly according to a defined process or sporadically. Half of the responders (50%) reported using Social Media as source of AEFI reports; access to mobile phones and internet may increase the importance of this channel to receive or have access to reported AEFIs. Only 24% of the respondents reported to have access to WHO Vigibase or FDA VAERS.^4^ Both databases are accessible for the public to screen for AEFIs. 62% of the respondents answered to have established channels to monitor spontaneously reported AEFIs from licensee or licensor, which shows that manufacturers with licensing agreements have Safety Data Exchange Agreements (SDEAs) in place that define the exchange of safety data.

### 3.3 Collection and handling of AEFIs from active safety monitoring

Only 23% of the respondents reported to use the Brighton case definitions of AEFIs, which are useful for the standardization of AEFIs and are required by the European Medicines Agency (EMA) in the signal management and risk assessment process of vaccines [12] (Fig. 3). 68% of the respondents use the CIOMS/WHO definitions for Vaccine PV which shows that the concept of Vaccine PV is implemented by these manufacturers [5]. 88% of the respondents collect safety data from clinical trials whereas 71% have SOPs in place on the handling of safety data from clinical trials, raising a point of discrepancy. Manufacturer must have the oversight over the trials and have SOPs in place that allow the delegation of tasks (CRO, etc.) [13]. Product Quality Complaints (PQCs) handling and management is a GMP requirement and thus all respondents have such procedures in place, and 76% of the respondents reported to have SOPs in place for the handling of PQCs. 59% reported having documented procedures in place to determine or confirm if the reported PQC includes an AEFI, which includes the assessment of potential harm (i.e., adverse events) [14]. 38% of the respondents have SOPs in place to perform a Health Hazard Evaluation (HHE) in the frame of GMP complaint investigation [15]. 88% of the responders indicated to have follow-up procedures in place in case the reported AEFI information is not complete, and 74% also have formal processes on how and when to close a case. Formal processes on follow-up procedures and case closure ensures that complete case information, as far as possible, is provided [16].

### 3.4 Management of AEFIs

All respondents (100%) have documented procedures (SOPs) in place that describe how to handle AEFIs, as well as SOPs to manage spontaneously reported AEFIs, and almost all respondents (97%) have SOPs on assessing seriousness. In contrast, 68% of the respondents have SOPs on medical review of the AEFIs (Fig. 4). 79% of the respondents indicate to have SOPs on assessing causality. Interestingly, more manufacturers have SOPs on causality than on medical review, though causality assessment requires medical review. The WHO algorithm for AEFI causality assessment can be used and handled technically; however, this algorithm does not replace causality assessment and medical review in the signal and risk management process [17]. Less than half of the respondents (44%) have access to the coding dictionaries. As per ICH, MedDRA coding of AEFIs is mandatory [18], however WHOart is still occasionally used by NRAs reporting AEFIs to WHO Uppsala Monitoring Center.

### 3.5 Management of aggregate (periodic) reporting (PSURs, annual reports, DSURs) and safety-related requests by regulatory bodies

91% of the respondents prepare PSURs of their marketed products, as the submission of aggregate reports, pre-marketing Annual Reports or development safety update reports (DSURs) and post-marketing periodic safety update reports (PSURs) is a mandatory requirement by regulatory authorities and supports the continuous surveillance of the product [19], [20]. 53% of the respondents prepare DSURs of their vaccines in development. In contrast, in previous questions, 88% of the respondents reported to collect SAEs and AEs from clinical trials and 71% have respective SOPs in place (see 3.3 above). 79% of the respondents have SOPs to prepare periodic/aggregate reports (e.g., PSURs, DSUR or Annual Reports) according to the NRA’s requirements, whereas 91% reported to prepare PSURs. Half of the respondents (50%) have SOPs for the preparation of specific safety reports requested by NRAs or WHO (Fig. 5). In 74% of the respondents the PV personnel performs safety-related reports.

### 3.6 Safety database systems

A safety database allows monitoring, assessing, and reporting to NRA safety information, as required by national legislation and its use has become best practice in PV. 62% of the respondents have a paper-based system and equally 62% use an Excel based system.

41% reported having an electronic safety database system and equally 41% use a commercial database system such as Aris Global, Argus, or a home-grown system, which facilitates for *state-of-the-art* AEFI case management and case handling but is not a regulatory requirement (Fig. 6).

29% of the respondents have the safety database system outsourced. The “yes” answers from the question on “Commercial database” and on “Outsourced database” add up to 70%, implying that almost ¾ of the respondents use a commercial electronic database. 29% reported having electronic system to support the PV activities (e.g., signal detection, monitoring of detected signals, tracking tools, metrics, etc.). 47% of the respondents have access to MedDRA; commercial safety databases (e.g. Aris Global/Argus) have MedDRA integrated in the system. More than 50% do not have access to MedDRA, in line with the answers concerning documented processes on MedDRA coding. MedDRA is a mandatory coding tool for all AEFI submissions in ICH countries [18]. 32% of the respondents use WHO Drug Dictionary which is not mandatory for vaccine manufacturers; however, this Dictionary is a helpful tool for organizations that also have drugs and/or for marketing authorizations in several different countries. 21% of the respondents have access to WHO Vigibase. The access is open for manufacturers to search for their own product, and Vigibase data mining can be a helpful tool in the signal management process [21].

### 3.7 Pharmacovigilance quality management system

A PV Quality Management System (PV QMS) is an integral part of the PV system. Such a PV quality system should cover the organizational structure, responsibilities, procedures, processes and resources, resource management, compliance management and record management. Almost all respondents have a summary list of all SOPs available (94%). 68% of the respondents have quality control (QC) processes included in their PV activities [22]. PV trainings should be provided at least once a year and given to new employees before they start their activities in the organization and 82% reported having regular training for PV personnel (Fig. 7). Each employee working in PV must have their training documents and their JD ready and up to date (signed and dated). 88% of the respondents have the training documented and archived; this is in alignment with the answers above and in Section 3.1. 73% indicated that they have procedures in place to perform internal PV audits, whereas in Section 3.1, 59% respondents indicated that they perform internal PV audits; 56% of the respondents have procedures to be followed in case of a NRA or WHO inspection. In Section 3.1, 35% indicated that their PV system has been inspected by a NRA or by WHO. More than half of the respondents are aware of the possibility of PV inspections and are ready for WHO inspections for prequalified vaccines. 82% of the respondents have procedures in place to perform corrective and preventive actions (CAPAs). CAPAs are the consequence of audit or inspection findings. The number of respondents having CAPA procedures in place is higher than the number of respondents having audit (73%) and inspection (56%) procedures in place. Perhaps these CAPA procedures refer to GMP rather than to PV specifically. 65% of the respondents have procedures to measure compliance for regulatory reporting of individual AEFIs or periodic/aggregate reports (metrics). Compliance measurements are a mandatory requirement from regulatory authorities and are subject to inspections [23].

### 3.8 Safety signal, risk Management, and safety communication

Safety signal management ensures that all available safety information are regularly screened for potential safety impact and change of the benefit-risk balance (Fig. 8). 62% of the respondents reported to perform signal detection activities, 59% have procedures to perform signal detection, and 56% have procedures to handle detected signals (validation, evaluation process etc.). 50% of the respondents reported having procedures to perform the risk assessment of an evaluated and confirmed signal. A risk management system includes the risk assessment of evaluated and confirmed signals as well as the monitoring of the outcome of measures to reduce risks to a minimum including a Risk Management Plan (RMP) [24], [25], [26]. 65% of the respondents reported having issued an RMP for a newly developed vaccine. 59% reported having medical experts at hand to continuously monitor the safety of their vaccine(s) throughout the product lifecycle. 68% reported having procedures to ensure that the safety information in the Reference Safety Information/labelling/package insert is up to date. 53% of the respondents reported to have procedures on how to communicate safety issues to stakeholders. The “CIOMS Guide to Vaccine Safety Communication” is a good reference for manufacturers to produce Vaccine Safety Communication Plans (including Templates) [27].

### 3.9 Pharmacovigilance agreements concerning exchange of safety data

To monitor the safety of vaccines and enable appropriate actions, when required, a manufacturer needs access to all available safety data of their vaccine. 76% of the respondents have agreements and respective procedures in place. Small companies without business partners and no exchange of safety information do not need such procedures (Fig. 9). 65% of the respondents have procedures in place for exchanging quality complaint data. In case of co-development, co-marketing, co-distribution, exchange of quality complaint data is a GMP requirement. 79% of the respondents have PV staff ensuring that SDEAs can be set up. Such agreements require experts from PV, RA, Manufacturing, QA and legal and must be regularly (e.g., annually) reviewed and updated in case of changes.

## 4 Discussion

The goal of this PV landscape analysis was to understand the existing PV structures, key areas and processes of vaccine manufacturers from emerging countries, on an institutional level. The results provide a tool for manufacturers to benchmark and serve as a basis to align their PV system with international expectations, and also for the Network to provide target training to strengthen vaccine safety monitoring, where needed. Due to the fact that many NRAs from emerging countries recently joined the ICH group (cf. https://www.ich.org/page/members), this landscape analysis provides an opportunity for alignment in manufacturers’ PV activities, as this survey was based on the essential ICH pharmacovigilance requirements for the pharmaceutical industry, e.g. supported by GVP (Good Vigilance Practice). The WHO Regulatory benchmarking tool includes these specifications.

The survey showed that most PV requirements, procedures and processes are implemented by vaccine manufacturers from emerging countries.

All manufacturers have reported having SOPs related to the handling of AEFI cases in place, and the vast majority have established channels to collect AEFI cases. Almost (97%) all respondents have procedures in place to define seriousness, whereas a lower proportion have SOPs on assessing causality and perform medical review of the individual cases (e.g., important for the assessment of seriousness, expectedness and causality), while less than half have access to MedDRA coding. The CIOMS /WHO definitions for vaccine PV are used by a third and the Brighton case definitions of vaccine specific AEFIs, an important tool for the standardization and harmonization within vaccine PV, are used by only a quarter of respondents. Vigibase would be a good database as NRAs from more than 130 countries transmit their adverse drug reaction reports to the WHO UMC (Uppsala Monitoring Center) that runs this global database which contains over 20 million reports. Screening this database could extract manufacturer vaccine’s AEFIs that they would not have received from NRA. However, this database is mainly designed for drugs which might limit its usefulness for vaccines. VAERS contains all AEFI reports to US FDA from world-wide sources. However, this database contains only US FDA-approved vaccines, thus limiting the benefits to use this channel by the emerging countries’ vaccine manufacturers. This database could however be a helpful tool in signal management, as it can provide information on reports from similar vaccines authorized in the US. These discrepancies indicate that although all respondents have SOPs to handle AEFI cases, in the case management and case assessment processes there is opportunity for higher implementation. Assessment of seriousness, expectedness, causality, and coding are key PV activities and should be performed and described in documented processes (SOPs).

An area for strengthening PV practices seems the handling of PQCs and reconciliation with AEFIs: 76% have procedures in place on PQC handling, but 59% have procedures to determine if a PQC is an AEFI and 38% on how to manage a situation where a PQC might have an impact on safety of the exposed population (e.g., Health Hazard Evaluation).

The analysis indicates that key PV activities such as PSURs, RMPs, signal and risk management as well as safety labelling and communication are performed by over 50% of the respondents, however some activities may be performed without underlying defined processes. Less than half of the respondents have internal structures for signal and risk management such as Vaccine Safety Teams or a Safety Governance Board. The results of the survey suggest that challenges for continuous safety monitoring may include periodic reporting of safety information, signal and risk management including risk communication and respective operational models.

Most of the respondents have elements of a PV QMS in place, such as a PV policy, a PSMF/DDPS, organizational charts, job descriptions, PV training programs, SOP library and dedicated PV personnel. Still, the survey indicated that less than 70% have PV QC procedures to measure compliance (metrics), and less than 60% perform internal PV system audits. Internal audit activities can significantly help to build a structure of continuous improvement of the PV System, and establish a PV System risk profile.

Specific safety database systems are not a legal requirement; however, as safety databases allow for a structured processing and evaluation of safety information, the use of safety databases has become best practice in PV. The safety database is the main repository for collected safety information, facilitates regulatory reporting (individual cases or aggregate reports) and is a key source of information for signal detection and ongoing evaluation of the benefit-risk profile, also important in building international collaborations. Although the results of the survey do not provide a deep understanding on how the manufacturers have set up their safety database systems, all respondents reported to have a safety database in place, either paper-based system supported by excel-lists or commercial database systems. Commercial database systems have the advantage that case processing steps (i.e., case entry, coding, narrative writing, medical review including assessment of expectedness, seriousness and causality, case follow-up, case closure, including QA and QC steps) are clearly defined and no step can be omitted. Still, paper-based/excel-supported systems may be used by small manufacturers with a limited number of AEFI reports annually. It is vital that the safety of all medicinal products, including vaccines is monitored throughout their life cycle. Manufacturers should have systems in place to fulfil safety responsibilities: (a) to ensure that all sources of information are regularly screened to identify potential signals, (b) to ensure that appropriate action is taken in response to evidence that impacts the benefit-risk balance, and (c) to keep regulatory authorities, HCPs and vaccinees informed in case of a change of the benefit-risk balance. These key activities of PV are mandatory regulatory (ICH) requirements.

Formal contracts and agreements with all third parties that may expose the third party to safety data should be available, and thus could form part of their PV system. Formal contracts should be in place when (a) outsourcing pharmacovigilance activities and using service providers (e.g., CROs) for PV activities, (b) using third party distributors and manufacturers (incl. e.g., distribution via UNICEF, and others), and (c) co-marketing, co-licensing or co-promotion of their vaccines. Such agreements can be a challenge when introducing new vaccines in different countries [28].

Industry is considered a key player to ensure the improvement of global vaccine safety [29]; therefore, manufacturers strive to implement international recognized best-practices complying with national, international or supranational requirements to become a reliable partner to regulatory bodies. The International Federation of Pharmaceutical Manufacturers & Associations (IFPMA) assessed PV practices and systems in 6 countries including high income and middle income countries, on how they have implemented processes to address some of the challenges identified in PV for biotherapeutic products [30]. Their report, from an industry perspective, also suggested strengthening PV through harmonization and training programmes.

In discussions with representatives of DCVMN members at the meeting reported here, their biggest challenge is the need for continuous, real-time access to safety data, and the receipt of comprehensive quality data is a high priority. To perform meaningful case analyzes and subsequent signal and risk management, real-time accurate and comprehensive safety information is necessary. Manufacturers would appreciate improved and regular access to comprehensive safety data from the national immunization programs.

## 5 Conclusion

The analysis provides a “bird's-eye view” of the structure of PV areas and the operational dimensions and activities covered by each area.

Overall, the survey shows that all participating manufacturers have established vaccine safety capacity, mainly in collecting AEFIs and assessing seriousness as well as implementing a number of standardized processes. The survey also shows that there are differences in the maturity of the PV system, of the PV QMS, of signal and risk management, as well as safety governance. These key components of a functional vaccine PV system, compliant with ICH standards warrant further support. WHO has made great efforts in establishing and strengthening AEFI reporting and PV training in emerging countries as reporting of AEFIs as well as trained PV personnel is a key component for a functional vaccine safety monitoring system [31], [32].

For further enhancement of the manufacturers’ PV system, to accelerate the global vaccine supply, DCVMN can support the vaccine manufacturers in three main areas, to continuously improve their PV Systems:

• Develop PV checklists to facilitate gap analysis and internal PV audits;
• Develop algorithms to facilitate the implementation of corporate safety governance;
• Coordinate discussions on PV best practices, databases for small and medium entities (SMEs) and support training in pharmacovigilance management for manufacturers in emerging countries.

Participants agreed that this report be prepared to share the data with those who were unable to join the workshop, and to share with the global health community for open dialogue.

## Declaration of Competing Interest

The authors declare that they have no known competing financial interests or personal relationships that could have appeared to influence the work reported in this paper.

## Footnotes

1 Developing Countries Vaccine Manufacturers Network, cf. www.dcvmn.org.

2 Argentina, Bangladesh, Brazil, China, India, Indonesia, Pakistan, Russia, S. Africa, S. Korea, Thailand, Taiwan, Vietnam.

3 www.dcvmn.org.

4 Vaccine Adverse Event Reporting System.
